# Real-Time Detection and 3D Localization of Coronary Atherosclerosis Using a Microwave Imaging Technique: A Simulation Study

**DOI:** 10.3390/s22228822

**Published:** 2022-11-15

**Authors:** Md Asiful Islam, John L. Volakis

**Affiliations:** 1 Department of Electrical and Electronic Engineering, Bangladesh University of Engineering and Technology (BUET), Dhaka 1205, Bangladesh; 2 College of Engineering and Computing, Florida International University, Miami, FL 33174, USA

**Keywords:** biomedical sensors, coronary atherosclerosis, human health monitoring, microwave imaging

## Abstract

Obtaining the exact position of accumulated calcium on the inner walls of coronary arteries is critical for successful angioplasty procedures. For the first time to our knowledge, in this work, we present a high accuracy imaging of the inner coronary artery using microwaves for precise calcium identification. Specifically, a cylindrical catheter radiating microwave signals is designed. The catheter has multiple dipole-like antennas placed around it to enable a 360° field-of-view around the catheter. In addition, to resolve image ambiguity, a metallic rod is inserted along the axis of the plastic catheter. The reconstructed images using data obtained from simulations show successful detection and 3D localization of the accumulated calcium on the inner walls of the coronary artery in the presence of blood flow. Considering the space and shape limitations, and the highly lossy biological tissue environment, the presented imaging approach is promising and offers a potential solution for accurate localization of coronary atherosclerosis during angioplasty or other related procedures.

## 1. Introduction

Coronary atherosclerosis is a deadly disease, yearly taking away millions of lives worldwide [1]. The majority of acute coronary events are precipitated by the rupture of a vulnerable atherosclerotic plaque in the coronary system, and subsequent thrombogenesis [2]. The key to plaque vulnerability is still obscure, even though recent advances in intravascular imaging technology have enabled the collection of a wealth of data on unstable atherosclerosis in all its stages of development [2], both in clinical and in ex vivo settings. Plaque type and morphology are relevant for intervention planning, and significantly affect long-term treatment outcome [2]. Hence, devising accurate and robust imaging technology is critical towards effective treatment strategies for coronary atherosclerosis.

Until now, intravascular ultrasound (IVUS) [3] and optical coherence tomography (OCT) [4] demonstrated their potential value in assessing plaque morphology and pathophysiology and generated optimism that intravascular imaging would enable the accurate detection of high-risk plaques likely to cause clinical events [2]. However, recent histology based studies and large-scale studies of coronary atherosclerosis revealed significant limitations of existing imaging modalities in detecting vulnerable plaque characteristics and high-risk lesions [2]. The miniaturization of medical devices and advances in image and signal processing permitted the development of novel modalities, e.g., near infrared spectroscopy (NIRS) [5], intravascular photoacoustic (IVPA) imaging [6], near infrared fluorescence (NIRF) imaging [7], and time-resolved fluorescence spectroscopy (TRFS) [8] that appear to be able to address certain limitations of either IVUS or OCT and provide additional information about plaque morphology and pathobiology. Nevertheless, no single existing technique enables a complete assessment of the plaque. To address this challenge, hybrid imaging has been suggested by researchers [2]. In this context, microwave imaging (MWI) can be a complementary choice as it has already demonstrated excellent contrast between healthy and anomalous tissue [9,10,11,12,13,14,15,16,17,18]. Notably, MWI would render added information to better resolve coronary plaque and help the treatment plan. We remark that microwave and mm-wave sensors have already shown their potential in different health monitoring applications [19,20,21].

In this work, the first ever demonstration of MWI’s capability was performed for real-time detection and localization of pulmonary atherosclerosis. Specifically, a cylindrical catheter was designed with multiple dipole antennas imprinted surrounding the entire outer surface of the catheter. For an unambiguous 3600 field-of-view, the plastic catheter has a central metallic rod running along its axis. A realistic numerical model of the artery was employed, and the catheter was placed inside the artery. Subsequently, full 3D image of the artery was reconstructed employing an imaging functional obtained after suitable modification from a recent work of the authors. Successful detection and localization of the plaques on the artery walls were performed from the visualization of the 3D images. Hence, the presented work can pave the way for successful deployment of MWI in the real-time imaging of coronary atherosclerosis.

## 2. Imaging Method

### 2.1. Catheter Design

**Figure 1:**
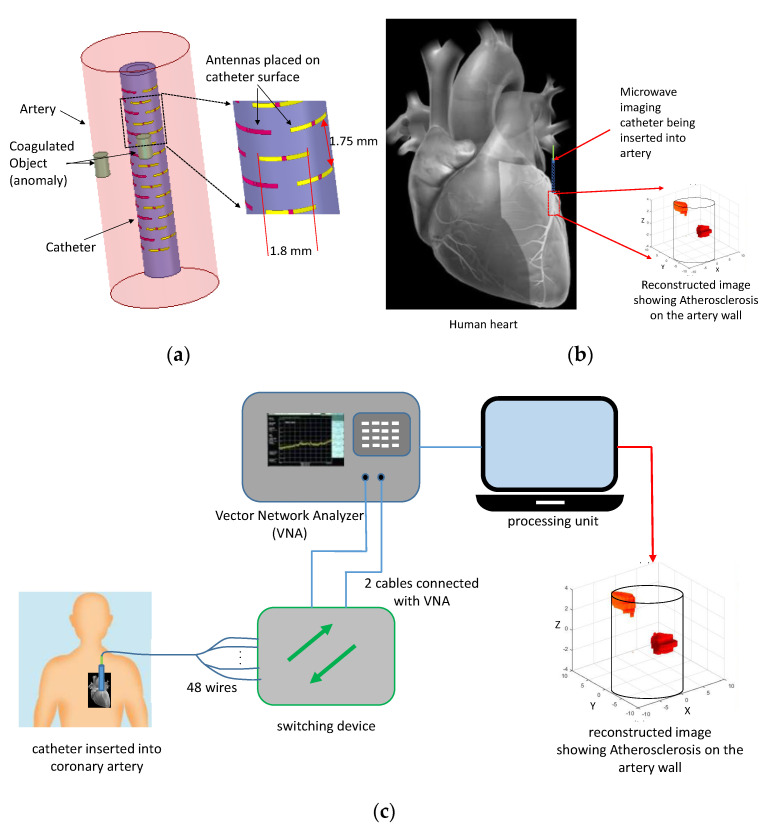
(**a**) The imaging catheter placed inside the coronary artery model, (**b**) overview of the imaging process, (**c**) envisioned block diagram of the complete system performing the imaging.

As shown in Figure 1, the proposed imaging device is a cylindrical catheter to be pushed through the coronary artery filled with blood. We model the artery as a circular cylinder with radius, $r_{a}=5\;\mathrm{mm}$. The cylindrical catheter to be inserted inside the artery has a radius of $r_{c}=1.5\;\mathrm{mm}$. The catheter is made of plastic material with permittivity, $\varepsilon_{r,c}=5$ and loss tangent, $tan\delta_{c}=0.001$. A metallic rod of radius 0.8 mm is inserted along the axis of the plastic catheter for a reason explained in Section 4. Overall, 48 curved-dipole antennas, each 1.8 mm long, are placed conformally on the outer surface of the catheter, as shown in Figure 1. Any anomaly (fat, calcium, etc.) coagulated on the artery wall will be continuously detected and localized from the images obtained using the data collected from these 48 dipoles. The data collection is carried out in a multi-static manner, i.e., sequentially exciting one of the antennas and measuring from all others. Each of the 48 dipoles is connected to the outer data acquisition circuit through a bundle of 48 wires as shown in Figure 1c. A standard block diagram of the complete coronary artery imaging system [18] is shown in Figure 1c. The scattering parameters data are measured by the two-port vector network analyzer (VNA). The transmit and receive antennas are selected sequentially using the switching device. Finally, the collected data are sent to the processing unit from the VNA to calculate and display the image. We remark here that the input power at the transmit antenna was 1 mW. As a result, the corresponding maximum Specific Absorption Rate averaged over 1 g of tissue was SAR1g,max ≈ 0.6 W/kg. This value is far below the limit 1.6 W/kg based on the IEEE C95.1-1999 [22] and FCC [23] safety exposure guidelines.

### 2.2. Imaging Process

As already mentioned, microwave imaging is a highly ill-posed problem, and it often requires time-consuming algorithms to obtain the image. These algorithms are not well-suited for this artery wall imaging problem as in this case we need a method that yields fast image recovery. Moreover, the conventional MWI problems deal with closed-domains, i.e., the antennas surround the imaging domain [9,10,11,12,13,14,15,16,17,18]. However, the problem of imaging the artery walls using an intravascular catheter is not a closed-domain scenario, see Figure 1b. Rather, in this case, the transceiver antenna locations are restricted to be on the catheter only. To address all these challenges, we employ a modified version of the recently proposed loss compensated back propagation (LC-BP) technique [22] by the authors. Notably, LC-BP was originally proposed for a closed-domain case to enable fast and accurate detection and localization of the anomaly inside highly lossy biological medium. In LC-BP, the imaging functional is given by [24]

(1) $$I_{2D}\left(x_{pix},y_{pix}\right)=\left|\sum_{q=1}^{Q}\sum_{\begin{array}{c} m=1,\\ m\neq q \end{array}}^{Q}\rho \rho^{\prime }G_{2D}^{*}\left(\rho ,\rho^{\prime },\varphi ,\varphi^{\prime }\right)\;S_{qm}^{s}\left(\rho^{\prime },\rho ,\varphi ,\varphi^{\prime }\right)\right|$$

where the 2D Green’s function, *G*_2*D*_ of the medium is given by,

(2) $$G_{2D}\left(\rho ,\rho^{\prime },\varphi ,\varphi^{\prime }\right)=\frac{\beta^{2}}{4\omega \varepsilon }\overset{n=+\infty }{\underset{n=-\infty }{\sum }}\left[H_{n}^{\left(2\right)}\left(\beta \rho^{\prime }\right)\frac{J_{n}\left(\beta a\right)}{H_{n}^{\left(2\right)}\left(\beta a\right)}H_{n}^{\left(2\right)}\left(\beta \rho \right)e^{jn\left(\varphi -\varphi^{\prime }\right)}\right]$$

Here, $S_{qm}^{S}$ is the scattered field when q-th antenna is excited and the signal is measured at the m-th antenna, $\;H_{n}^{\left(2\right)}\left(\cdot \right)$ refers to n-th order Hankel function of the second kind, $J_{n}\left(\cdot \right)$ refers to n-th order Bessel function, ω is the frequency in rad/s, $\varepsilon =\varepsilon_{0}\varepsilon_{r}$ is the permittivity of the medium, and β = 2π/λ is the propagation constant of the medium. In addition, Q is the total number of antennas placed on the cylindrical catheter, $\left(x_{pix},y_{pix}\right)$ is the coordinate position of the pixel to be imaged, ρ and $\rho^{\prime }$ are the distances from the pixel to the transmitting and receiving locations, respectively.

**Figure 2:**
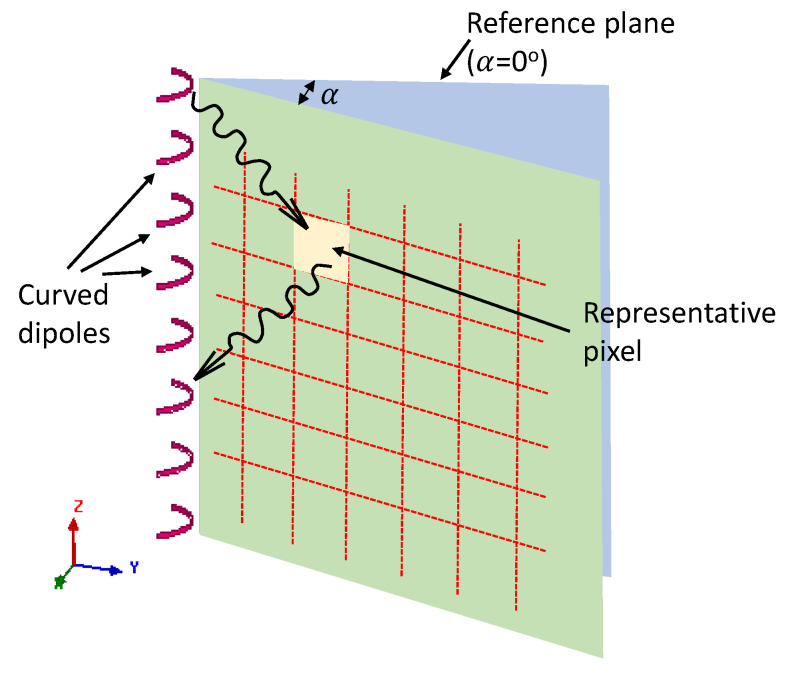
Imaging carried out on $\varphi =\alpha \;(0^{{}^{\circ}}\leq \alpha <360^{{}^{\circ}})$ planes.

The imaging functional of (1) was originally employed to obtain image on a 2D plane perpendicular to the dipole antenna orientation (TM polarization) [24]. However, to obtain a full 3D image for the cylinder-shaped artery domain shown in Figure 1, modifications of the imaging functional given in (1) have to be brought about. These modifications are required for two reasons: (1) to incorporate the change in antenna gain with respect to varying pixel positions in the 3D space, (2) to obtain the E-field component that fulfils the TM polarization condition (E-field ⊥ image cut plane). To address the first modification, we consider that the 3D imaging domain is composed of a number of 2D planes with $\varphi =\alpha \;(0^{{}^{\circ}}\leq \alpha <360^{{}^{\circ}})$ (*ρz* planes where *ρ* is the distance from *z*-axis), as shown in Figure 2. On each of these 2D planes, the pixel intensities (image) are to be calculated. To do this, we introduce two antenna radiation pattern factors, $g_{T}$ and $g_{R}$ for the transmit and receive antennas, respectively, in (1) to account for the pattern variations for each pixel. For the second modification, we introduce two polarization factors, ${\left[\cos\left(\alpha_{1}\right)\;\right]}_{T}^{g1}$ and $\;{\left[\cos\left(\alpha_{2}\right)\;\right]}_{R}^{g2}$ in (1). We remark that these factors will take maximum value (equal to unity) whenever the pixel to be imaged is located on the reference plane ($\alpha =0^{{}^{\circ}}$) shown in Figure 2. After introducing the above factors, we obtain the image at a pixel given by

(3) $$I_{3D}\left(x_{pix},y_{pix},\;z_{pix}\right)=\left|\overset{Q}{\underset{q=1}{\sum }}\overset{Q}{\underset{\begin{array}{c} m=1,\\ m\neq q \end{array}}{\sum }}\begin{array}{c} \rho \rho^{\prime }G_{2D}^{*}\left(\rho ,\rho^{\prime },\varphi ,\varphi^{\prime }\right)\;S_{qm}^{s}\left(\rho^{\prime },\rho ,\varphi ,\varphi^{\prime }\right)g_{T}\left(\rho ,\varphi ,z\right)g_{R}\left(\rho ,\varphi ,z\right)\\ \;\;\;\;\;\;\;\;\;\;\;\;\;\;\;\;\;\;\;\;\;\;\;\;\;\;\;\;\;\;\;\;\;\;\;\;\;\;\;\;\;\;\;\;\;\;\;\;\;\;\;\;\;\;\;\;\;\;\times \left|{\left[\cos\left(\alpha_{1}\right)\;\right]}_{T}^{g1}{\left[\cos\left(\alpha_{2}\right)\;\right]}_{R}^{g2}\right| \end{array}\right|$$

where, $g_{T}$ = the E-field radiation pattern of the transmit antenna,

$g_{R}$ = the E-field radiation pattern of the receive antenna,

$\alpha_{1}\;$ = the azimuth angle between the plane perpendicular to the transmit dipole and the plane where the pixel is located,

$\alpha_{2}\;$ = the azimuth angle between the plane perpendicular to the receive dipole and the plane where the pixel is located,

*g*1, *g*2 = factors to adjust the cosine polarization factors.

As pointed out in [24], suitable coordinate transformation has to be performed every time (3) is employed to ensure the center of each pixel coincides with the center of the coordinate system.

## 3. Data Generation and Imaging Results

As already mentioned, we modeled the artery as a circular cylinder. All dipoles are identical curved copper sheets with the feed point in the center of the two arms (see Figure 1a). They are resonant at the operating frequency of 6 GHz. The artery was filled in with blood whose permittivity and loss tangent were set to, $\varepsilon_{r,b}=52.18$ and $tan\delta_{b}=0.39$, respectively, at 6 GHz obtained from [25,26]. Two cylindrical anomalies (possible coagulated calcium/fat) of radius 1 mm and height 2 mm were placed near the artery wall at two different places and subsequent data collection was carried out for three-dimensional image reconstruction using (3). The anomaly permittivity and loss tangent were set to, $\varepsilon_{r,a}=4.94$ and $tan\delta_{a}=0.19$, respectively, as they are the typical values for fat at 6 GHz [25,26].

The entire imaging set-up–the artery along with the catheter with curved dipoles on its outer surface–is designed in Ansys HFSS, and the synthetic data were obtained using its full-wave simulations. The data thus obtained are corrupted with additive white Gaussian noise to obtain a signal-to-noise ratio (SNR) of 20 dB, typically employed for microwave image reconstructions [9]. The image intensity is normalized between 0 to 1 after setting the intensity value under 0.2 to zero [27].

### 3.1. Justification of Using Metallic Rod in the Catheter (Resolving ‘Image Ambiguity’)

**Figure 3:**
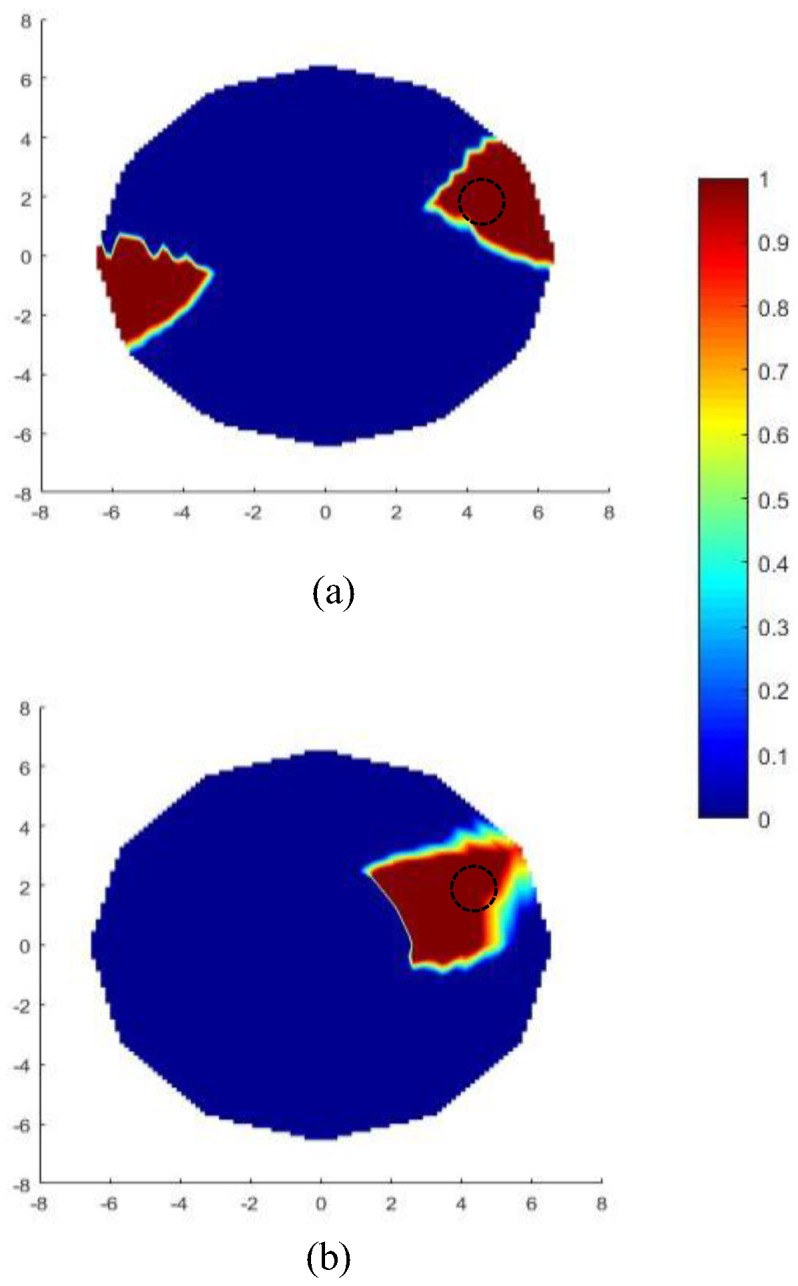
An example reconstructed image on *xy* (z = 0) plane: (**a**) image without metallic rod (ambiguity present), (**b**) image with metallic rod inserted (no ambiguity). Dotted circle shows the actual position of the anomaly.

**Figure 4:**
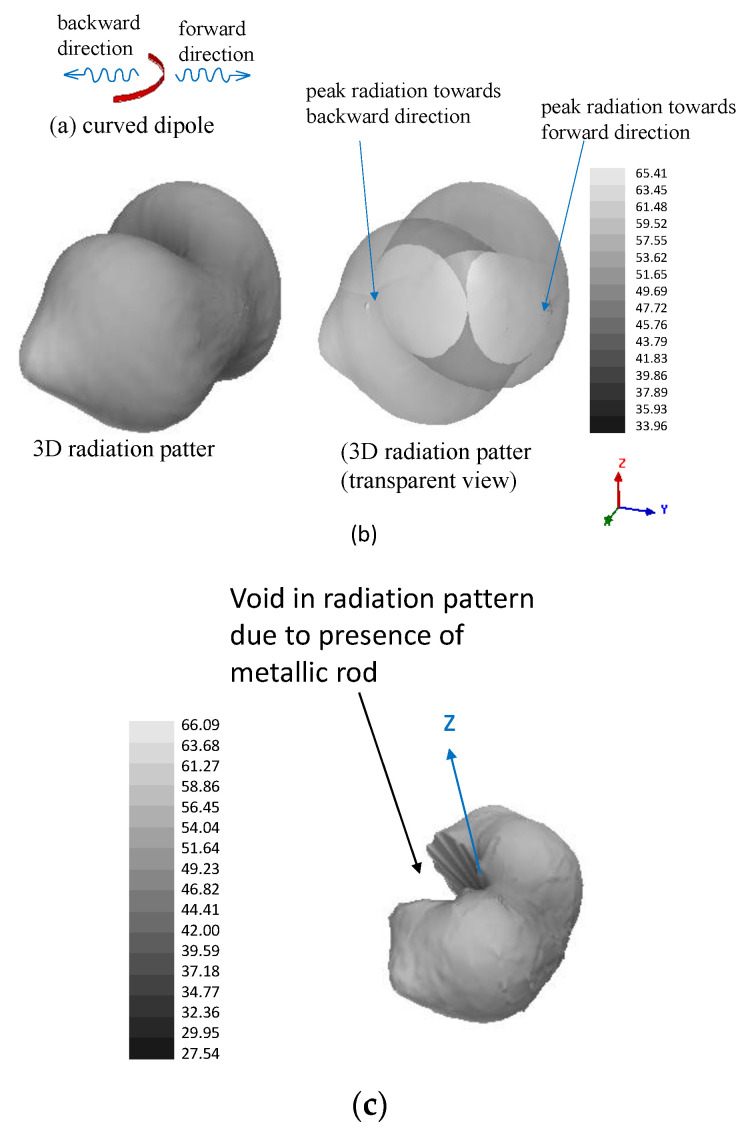
(**a**) Curved dipoles showing the forward and backward propagation direction, (**b**) E-field radiation pattern without the metallic rod, (**c**) E-field radiation pattern after the metallic rod is inserted.

If no metallic rod is employed inside the catheter, it was observed that the reconstructed image generates a false anomaly at the 1800 opposite location (and around) of the actual one on the xy plane (see Figure 3a). This is attributed to the radiation pattern of the curved dipole shown in Figure 4b (no metallic rod). As seen from this pattern, the dipole can also transmit (and receive) signals coming from its backward direction (see Figure 4a). Hence, Equation (3) would not be able to resolve whether the scattered signal is coming from the backward or forward direction and will show high intensity on both the actual and opposite location of the anomaly. This issue, referred to as ‘image ambiguity’, can simply be resolved by inserting a metallic rod along the axis of the catheter as already mentioned in Section 2. This metallic rod will block the scattered signals coming from the opposite side of the actual anomaly location, which is evident from the radiation pattern shown in Figure 4c. After inserting the rod, the reconstructed image is shown in Figure 3b where no false anomaly location is being shown.

### 3.2. Imaging Scenario and Results

**Figure 5:**
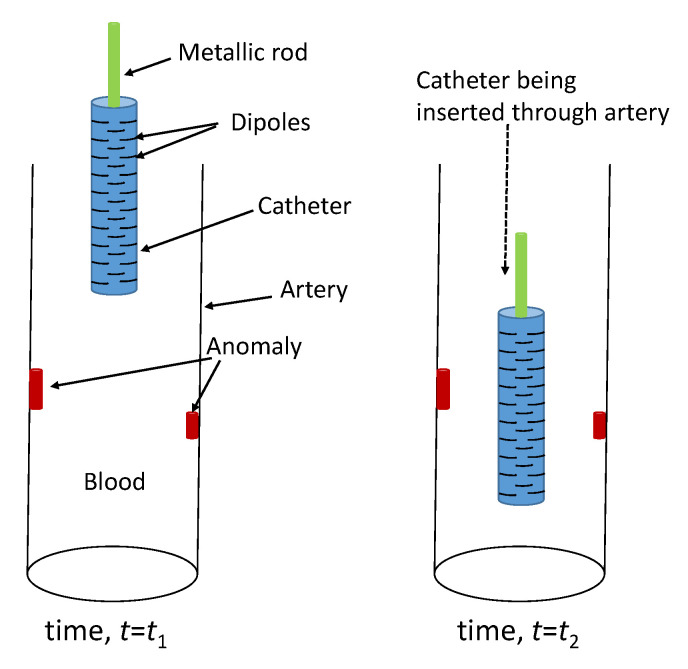
Two sets of measurements, *S*_1_ and *S*_2,_ are being taken at times, *t* = *t*_1_ and *t* = *t*_2_, respectively.

**Figure 6:**
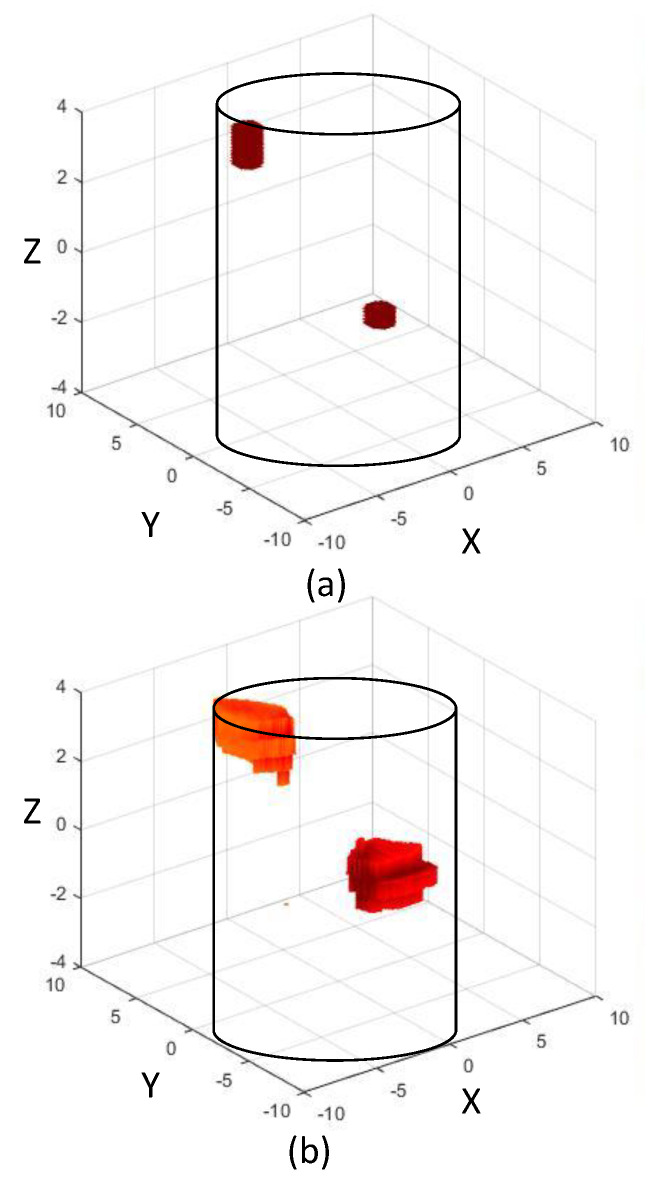
(**a**) Actual anomalies shown inside the 3D cylindrical imaging domain, (**b**) Reconstructed anomalies shown inside the 3D cylindrical imaging domain.

**Figure 7:**
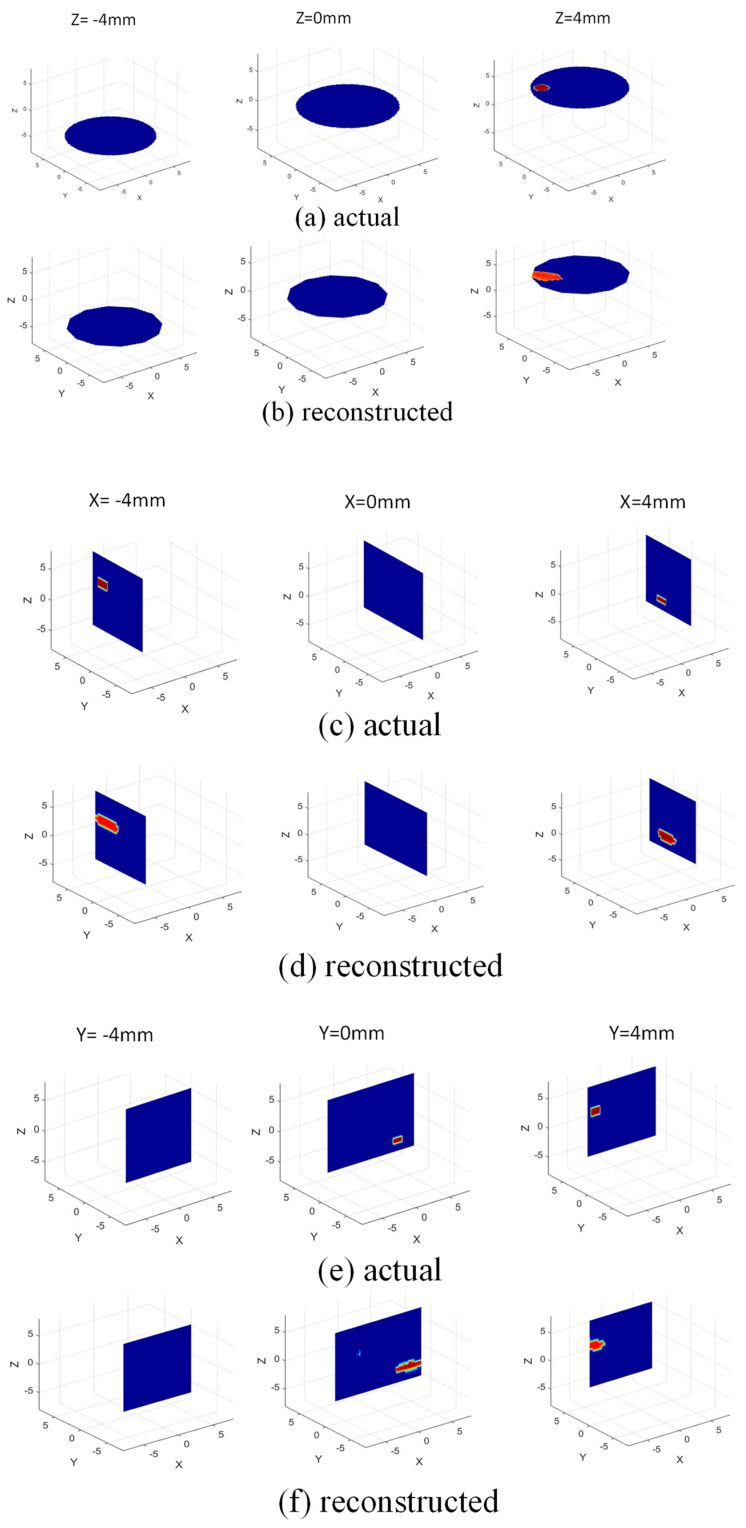
3D image on different 2D planes: (**a**,**b**) actual and reconstructed image on *xy* planes (*z* = −4, 0, 4 mm); (**c**,**d**) actual and reconstructed images on *yz* planes (*x* = −4, 0, 4 mm); (**e**,**f**) actual and reconstructed images on *zx* planes (*y* = −4, 0, 4 mm), respectively.

Next, we explore a typical imaging scenario where two locations of fat accumulation (anomalies) are present on the inside wall of an artery. The cylindrical anomalies are located at (−4 mm, 3 mm, 4 mm) and (4 mm, 1.5 mm, −2 mm) and have radius = 1 mm, 1 mm and height = 2 mm, 1 mm, respectively. The catheter is inserted into the artery and slowly pushed down through it, while measurements are being recorded from the dipoles continuously at different time instances. We assume a scenario where, at two time instances t1 and t2 (see Figure 5), we have two sets of measurements S1 and S2, respectively. At time t1, the anomalies are out of the imaging zone, hence, S1 measurements would only ‘see’ the artery walls (without anomalies). However, at time t2, the anomalies are present within the imaging zone, hence S2 measurements will ‘see’ the anomalies located on the artery walls. To obtain the scattered field, $S^{S}$ to be employed in (3) to carry out the image reconstruction, we get $S^{S}$ = S1 − S2. The reconstructed image is thus a differential image, which shows the locations of the anomalies present on the artery walls. In Figure 6b, the reconstructed anomalies are shown inside the cylindrical artery, along with their actual size and location presented in Figure 6a. For a complete understanding of the image reconstruction on different planes taken from the full 3D reconstructions, Figure 7 is presented, where the image is shown on several *xy*, *yz*, and *zx* planes. By examining these, one can conclude that the anomalies on the artery walls are reliably detected and localized on all the planes of the 3D imaging domain.

## 4. Conclusions

The imaging of calcium deposit on the coronary artery walls is essential to successful treatment and preventive medical care. To this end, microwave tomographic techniques using a catheter can be an effective real-time imaging approach. However, traditional MWI imaging relies on a closed domain with the radiators/receivers placed surrounding the imaging domain. In contrast, the proposed approach in this work employs dipoles imprinted on a cylindrical catheter that radiate outwardly towards the artery walls, surrounding the catheter itself. To do so, a previously developed real-time imaging algorithm is modified and adapted. The key to the modification is the shielding of radiations on the catheter walls to ensure that only half of the artery walls are illuminated. Thus, false images are avoided, and accurate pinpointing of the calcium is made possible. Moreover, the use of simple dipoles as the microwave transceivers allows for future fabrication and measurements, likely for this geometry of small dimensions. Based on the presented imaging performance, it is anticipated that MWI can be a potential alternative or complementary technology to the existing ones for the real-time imaging of coronary atherosclerosis.

## Data Availability

Not Applicable.
